# Assessment of Primary Healthcare Providers’ Knowledge and Practices in Addressing Asymptomatic Hyperuricemia and Gout in the Asir Region of Saudi Arabia

**DOI:** 10.7759/cureus.51745

**Published:** 2024-01-06

**Authors:** Ibrahim Tawhari, Omar A AlQahtani, Mansour S Alqahtani, Rayan M Alshehri, Mohammed A Alsairy, Saad M Alqahtani, Abdulaziz M Aljaber, Mubarak M Alshahrani, Ali M Alqahtani, Mohammed A Alahmary, Nassir M Alsuairi

**Affiliations:** 1 Internal Medicine, College of Medicine, King Khalid University, Abha, SAU; 2 Medicine, College of Medicine, King Khalid University, Abha, SAU; 3 Medicine, College of Medicine, King Faisal University, Al-Ahsa, SAU; 4 Medicine, College of Medicine, University of Bisha, Bisha, SAU

**Keywords:** serum uric acid level, continuing medical education, primary healthcare providers, gout, asymptomatic hyperuricemia

## Abstract

Introduction and aim

Gout, the most common form of inflammatory arthritis, arises from hyperuricemia, a condition where elevated levels of uric acid lead to the deposition of monosodium urate (MSU) crystals in the joints. Nevertheless, it's important to note that not all cases of hyperuricemia result in gout.

Methodology

This cross-sectional study was conducted in the Asir region of Saudi Arabia, targeting primary healthcare physicians (PHPs) specializing in family medicine and general practice. The study utilized a modified electronic questionnaire, inspired by similar studies and aligned with recent guidelines, to assess PHPs' knowledge and practices concerning asymptomatic hyperuricemia (AH) and gout. The questionnaire encompassed the PHPs' demographic data and their knowledge and practices for AH and gout management.

Results

Out of 201 participating PHPs, the majority were male (68.2%), predominantly aged 25-34 years (73.1%), and practicing as general practitioners (61.2%). A significant proportion of PHPs had less than five years of experience (63.7%). In terms of education, 36.8% attended continuing medical education (CME) on AH or gout, and 66.7% were aware of the related management guidelines. The study revealed that the total knowledge score among PHPs averaged 5.18 out of seven, indicating a moderate level of knowledge. However, their practice level was moderate, with a mean practice score of 6.75 out of 12. The study also found no significant differences in knowledge scores based on gender, age, or years of experience, but significant variations were noted based on medical specialty.

Conclusion

There is a moderate level of knowledge and practice among PHPs in managing AH and gout in the Asir region. Despite adequate knowledge levels, there appears to be a gap in implementing this knowledge into practice, particularly in long-term management strategies. The findings emphasize the need for ongoing medical education and specialized training programs to bridge these gaps. The study provides a valuable framework for identifying and addressing similar challenges in other regions and medical practices.

## Introduction

Hyperuricemia is characterized by elevated serum urate levels higher than 7.0 mg/dl in males and 6.0 mg/dL in females [1]. Asymptomatic hyperuricemia (AH) is without the clinical manifestations of monosodium urate (MU) crystal deposition disease and stands as a significant medical concern. This condition, often interlinked with hypertension, chronic kidney disease (CKD), cardiovascular disease, and insulin resistance, harbors the potential to evolve into gout, a prevalent form of inflammatory arthritis, more common in men than women [2]. The incidence of gout varies globally, affected by demographic factors and research methodologies, with prevalence rates ranging from less than 1% to 6.8% [3]. The progression from AH to gout, which constitutes 90% of hyperuricemia cases, underscores the criticality of early detection and adept management by primary healthcare providers (PHPs) [4].

This study, conducted in the Asir region of Saudi Arabia, aims to dissect and elevate the understanding and practices of PHPs in managing AH and gout. The urgency of this endeavor is underscored by the looming risk of gout's painful and disabling consequences, which can significantly impair the quality of life and heighten the burden of associated health complications. Gouty arthritis, a direct outcome of MU crystal accumulation in joints due to elevated plasma uric acid levels, occurs when serum urate surpasses 6.8 mg/dL [5]. Notably, AH is not a standalone disease but a harbinger of advanced gout and a predictor of cardiovascular risks and imminent flares.

The etiological landscape of gout reveals that in 90% of patients, renal underexcretion of uric acid is the primary cause, while overproduction of purine accounts for the remaining 10%. Thus, the study's focus extends to identifying knowledge and practice gaps among PHPs. Such insights are instrumental in developing targeted educational initiatives and specialized training programs. These interventions aim to refine the PHPs' capabilities, ultimately elevating the caliber of care delivered to patients grappling with these conditions.

In 2018, a cross-sectional study was conducted among PHPs in Jeddah, Saudi Arabia, to assess their knowledge and practice levels of AH. The research found that only 32.8% of participating physicians had adequate knowledge about AH. In terms of clinical practice, the majority (88.1%) correctly advised patients to adopt a low-purine diet and healthy lifestyle changes. Still, nearly half incorrectly prescribed urate-lowering medication, and 10.9% incorrectly prescribed non-steroidal anti-inflammatory drugs (NSAIDs) [6]. According to Alraqibah et al. [7], the knowledge and practices of PHPs in the Qassim region of Saudi Arabia in the management of AH and gout were good (45.9%). Still, their level of practice could have been better by 23.3% [7]. Another cross-sectional study was conducted in India to evaluate physicians' knowledge, attitudes, and behaviors about hyperuricemia management. It was determined that 66.1% (230) out of 350 responses from physicians had adequate knowledge scores, while the remaining 33.9% (118) had inadequate scores [8].

The objective of this study is to evaluate the knowledge and current practices of PHPs in the Asir region concerning the management of AH. Additionally, the study aims to assess the treatment strategies used for patients with AH and gout. Furthermore, the study seeks to investigate the limitations clinicians face in adhering to current recommendations, taking into account the patient's medical history, to diagnose gout and determine the appropriate timing to initiate urate-lowering therapy (ULT).

## Materials and methods

Study design

A cross-sectional survey-based study was conducted in the Asir region of Saudi Arabia, focusing on PHPs specializing in family medicine and general practice. We excluded PHPs outside of the Asir region, as well as rheumatologists and internal medicine physicians, including residents and specialists. All PHPs in the Asir region were eligible to participate, and their responses to our electronic questionnaire formed the study population.

Data collection plan

We designed a modified electronic questionnaire for this study. Our study utilized a questionnaire originally designed and validated by Alraqibah et al. [7], which had been previously employed in their study on the knowledge and practice of primary healthcare providers in the management of asymptomatic hyperuricemia and gout in the Qassim region of Saudi Arabia. Permission to employ and modify the questionnaire for the current research was sought and granted by the corresponding author. To manage and analyze the data, we employed IBM SPSS Statistics software version 27.0.1 (IBM Corp., Armonk, NY).

Ethical considerations

We prioritized ethical considerations by obtaining informed consent from participants, clearly outlining the study's purpose and their rights regarding confidentiality and withdrawal. Each participant received a unique code number to safeguard their privacy during the analysis. No incentives or benefits were provided to participants. Before commencing the study, we secured approval from the Institutional Review Board (IRB) of King Khalid University, Abha, Saudi Arabia, which granted ethical approval for this study (approval number: ECM#2023-2403).

Scoring

We evaluated the overall knowledge of PHPs regarding the management of AH and gout using five questions. Correct answers were assigned a score of one while incorrect answers received a score of 0. Question five allowed for multiple correct responses, resulting in a total of seven knowledge items. The total knowledge score ranged from 0 to seven, where a higher score indicated a greater level of knowledge. We established a cutoff point for the total score to determine knowledge levels: less than 50% indicated poor knowledge, 50%-75% denoted moderate knowledge, and more than 75% represented a good level of knowledge. Assessing the practices of PHPs in managing AH and gout involved an eight-item questionnaire, where correct answers were assigned a score of one and incorrect answers received a score of 0. Items two and three consisted of three correct answers each, resulting in a total practice item score of 12. The total practice score ranged from 0 to 12, with a higher score reflecting a higher level of practice. We established a cutoff point for the total score to determine practice levels: less than 50% indicated poor practice, 50%-75% signified moderate practice, and more than 75% indicated good practice.

Statistical analysis

Both descriptive and inferential statistical analyses of the data were carried out. Quantitative variables were presented as mean ± standard deviation, while qualitative variables were expressed as percentages and numbers. The Mann-Whitney U test and the Kruskal-Wallis test were employed to compare knowledge and practice scores with sociodemographic characteristics. We confirmed the abnormal distribution of both knowledge and practice scores through normality tests using the Shapiro-Wilk test and the Kolmogorov-Smirnov test, necessitating the application of nonparametric tests. To determine the correlation between knowledge and practice scores, we used Spearman's rank correlation coefficient. A p-value <0.05 was considered statistically significant.

## Results

This cross-sectional survey evaluated 201 PHPs. Their sociodemographic characteristics are detailed in Table 1.

**Table 1:** Sociodemographic characteristics of the primary healthcare providers in the study group (n=201)

Study variables	N (%)
Age group	
25–34 years	147 (73.1%)
35–44 years	40 (19.9%)
45–54 years	13 (6.5%)
55–64 years	1 (0.5%)
Gender	
Male	137 (68.2%)
Female	64 (31.8%)
Medical specialty	
General practice	123 (61.2%)
Family medicine	78 (38.8%)
Years of experience	
<5 years	128 (63.7%)
>10 years	35 (17.4%)
5–10 years	38 (18.9%)
Attended continuing medical education (CME) on asymptomatic hyperuricemia (AH) or gout	
Yes	74 (36.8%)
No	127 (63.2%)
Read about AH or gout in the last year	
Yes	142 (70.6%)
No	59 (29.4%)
Aware of guidelines on the management of AH or gout	
Yes	134 (66.7%)
No 67 (33.3%)	

The majority were male (68.2%), with the most common age group being 25 to 34 years (73.1%). Regarding medical specialty, 61.2% were general practitioners, and 38.8% specialized in family medicine. In terms of experience, a significant portion of the PHPs (63.7%) had less than five years of experience, while 17.4% had more than 10 years.

The overall mean knowledge score recorded was 5.18 (SD: 1.41), with poor (22, 10.9%), moderate (92, 45.8%), and good knowledge (87, 43.3%) between participants, respectively (Table 2).

**Table 2:** Assessment of primary care providers’ knowledge in the management of asymptomatic hyperuricemia and gout (n=201)

Statement		N (%)
Which of the following values is correct regarding asymptomatic hyperuricemia (AH)?		
Serum uric acid level is >7 mg/dl in males and >6 in females		156 (77.6%)
Serum uric acid level is >6 mg/dl in both males and females		25 (12.4%)
Serum uric acid level is >6 mg/dl in males and >7 in females		20 (10.0%)
Uric acid precipitation causes an inflammatory response		
Correct		173 (86.1%)
Incorrect		13 (6.5%)
I do not know		15 (7.5%)
Asymptomatic hyperuricemia always progresses to gouty arthritis		
Correct		59 (29.4%)
Incorrect		124 (61.7%)
I do not know		18 (9.0%)
Asymptomatic hyperuricemia always needs treatment		
Correct		41 (20.4%)
Incorrect		143 (71.1%)
I do not know		17 (8.5%)
Which of the following is correct regarding the pathogenesis of AH?†		
Increase production of urate		152 (75.6%)
Decrease in renal excretion of urate		146 (72.6%)
Dietary source		148 (73.6%)
Increase renal excretion of urate		13 (6.5%)
Total knowledge score (mean ± SD)		5.18 ± 1.41
Level of knowledge		
Poor		22 (10.9%)
Moderate		92 (45.8%)
Good (43.3%)	87	

Table 3 presents survey data from PHPs on managing gout and AH.

**Table 3:** Assessment of primary care providers’ practices in the management of asymptomatic hyperuricemia and gout (N=201)

Statement	N (%)
Which of the following would you prefer to perform in the acute setting of the first gouty attack? (Multiple answers allowed)	
Joint aspiration	63 (31.3%)
Serum urate level	104 (51.7%)
Urate-lowering therapy	36 (17.9%)
None of the above	60 (29.9%)
Which of the following are effective during acute management?	
Colchicine	104 (51.7%)
Steroids	70 (34.8%)
Non-steroidal anti-inflammatory drugs (NSAIDs)	172 (85.6%)
None of the above	0 (0.0%)
Urate-lowering therapy should be started in which of the following? (Multiple answers allowed)	
Recurrent flares	147 (73.1%)
Asymptomatic hyperuricemia	54 (26.9%)
Tophi	95 (47.3%)
Radiographic findings	76 (37.8%)
What is the target serum uric acid after starting urate-lowering therapy?	
<3 mg/dl	10 (5.0%)
<10 mg/dl	12 (6.0%)
<6 mg/dl	137 (68.2%)
<8 mg/dl	42 (20.9%)
Anti-inflammatory prophylaxis can be continued for how long?	
<2 months	16 (8.0%)
1 month	38 (18.9%)
3-6 months	63 (31.3%)
No prophylaxis	84 (41.8%)
Which one of the following urate-lowering therapies is recommended over others?	
Allopurinol	185 (92.0%)
Febuxostat	12 (6.0%)
Probenecid	4 (2.0%)
Which of the following should be done if serum uric acid cannot be achieved by allopurinol?	
Add uricosuric lesinurad	30 (14.9%)
Increase the dose of allopurinol	76 (37.8%)
Switch to febuxostat	65 (32.3%)
Wait	30 (14.9%)
Which of the following regarding the modification of diet and lifestyle in case of gout/hyperuricemia do you practice in your clinic?	
Important, and I discuss it with patients in the clinic	168 (83.6%)
It is important, but I do not have time to discuss it with patients in a clinic	23 (11.4%)
Not important	5 (2.5%)
Not part of my medical consulting	5 (2.5%)
Total practice score (mean ± SD)	6.75 (±1.91)
Level of practice	
Poor	59 (29.4%)
Moderate	124 (61.7%)
Good	18 (9.0%)

Key practices were as follows: 31.3% used joint aspiration; 51.7% measured serum urate levels; and 17.9% initiated urate-lowering therapy during acute gout attacks. For acute management, non-steroidal anti-inflammatory drugs (NSAIDs) were preferred by 85.6%, colchicine by 51.7%, and steroids by 34.8%. Urate-lowering therapy was widely recommended for recurrent flares (73.1%), and the most common therapy goal was achieving a serum uric acid level below 6 mg/dl (68.2%). Anti-inflammatory prophylaxis opinions varied, with 41.8% advising no prophylaxis. Allopurinol was the preferred urate-lowering therapy (92%), and a purine-free diet and lifestyle modifications were considered important by 83.6% of PCPs. The overall practice quality was mixed, with 29.4% rated poor, 61.7% moderate, and 9% good.

**Figure 1:**
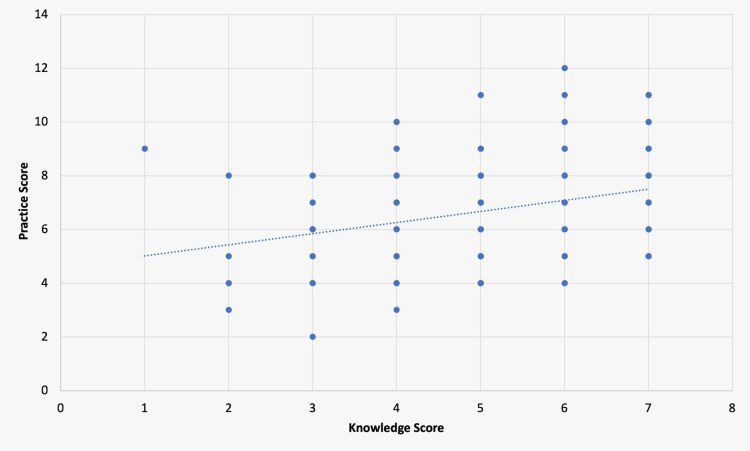
Spearman's correlation between the knowledge score and practice score

A significantly positive correlation between the knowledge and practice scores (r = p<0.001) can be observed in Figure 1.

Therefore, this indicates that the practice score is likely to rise whenever the knowledge score does.

Table 4 shows female participants averaged slightly higher in both knowledge (5.27) and practice (6.83) scores compared to their male counterparts (5.15 in knowledge and 6.71 in practice), although the difference wasn't statistically significant.

**Table 4:** Differences in the scores of knowledge and practice according to the sociodemographic characteristics of primary healthcare providers (n=201) p<0.05: significant; Mann-Whitney Z-test has calculated ZP value; Kruskal-Wallis H-test has calculated the HP value

Category	Subcategory	Knowledge score (7)		Practice score (12)	
		Mean	Standard deviation	Z/H-test; p-value	Mean
Gender					
	Female	5.27	1.37	Z=0.420; p=0.675	6.83
	Male	5.15	1.44		6.71
Age (in years)					
	25-34	5.04	1.45	H=6.721; p=0.081	6.73
	35-44	5.48	1.22		6.85
	45-54	5.77	1.36		6.31
	55-64	7.00	-		11.00
Medical specialty					
	Family medicine	5.54	1.29	Z=2.669; p=0.008*	7.37
	General practice	4.96	1.45		6.35
Years of experience					
	<5	5.06	1.48	H=3.433; p=0.180	6.61
	>10	5.60	1.19		6.94
	5-10	5.21	1.34		7.03
Attending continuing medical education (CME) on asymptomatic hyperuricemia (AH) or gout					
	No	5.08	1.50	Z=1.127; p=0.260	6.63
	Yes	5.36	1.24		6.95
Read about AH or gout in the last year					
	No	5.00	1.36	Z=1.158; p=0.247	6.44
	Yes	5.26	1.43		6.87
Awareness of guidelines on the management of AH or gout					
	No	4.61	1.36	Z=3.981; p<0.001*	6.39
	Yes	5.47	1.36		6.93

Knowledge and practice scores varied with age. The 55-64 year age group showed the highest scores (7.00 in knowledge, 11.00 in practice), while the 45-54 year age group had moderately high knowledge scores (5.77) but lower practice scores (6.31). The differences among age groups were not statistically significant. Family medicine practitioners scored higher in both knowledge (5.54) and practice (7.37) than general practitioners (4.96 in knowledge, 6.35 in practice), with a significant difference noted in the knowledge scores (Z=2.669; p=0.008). Practitioners with over 10 years of experience had higher scores (5.60 in knowledge, 6.94 in practice) compared to those with less than five years (5.06 in knowledge, 6.61 in practice). The differences were not statistically significant. Participants who attended CME on AH or gout or read about these topics generally scored higher in both knowledge and practice compared to those who didn't, though these differences weren't statistically significant. Those aware of guidelines on managing AH or gout scored significantly higher in knowledge (5.47 vs. 4.61) and practice (6.93 vs. 6.39) than those who were not, with a statistically significant difference in knowledge scores (Z=3.981; p<0.001).

## Discussion

This study was carried out to determine PHPs' knowledge and practices in managing AH and gout. The questionnaire questions were obtained from recent guidelines. The results of the study provide valuable insights into the knowledge and practice of PHPs in the management of AH and gout. The study found a majority correctly identified the definition of AH, but some confusion was evident. The PHPs demonstrated a good understanding of AH's pathogenesis. In contrast, Alraqibah et al.'s study in 2021 revealed that while most physicians in the Bisha province had good knowledge, their practice levels were concerning, with only 43.9% demonstrating good practice [7]. In China, Liu et al. [9] found that general practitioners had lower knowledge levels regarding gout compared to PHPs. Only 6.5% had a good understanding of gout, and the basic knowledge level was 55.6%. There was an awareness that AH does not always lead to gout and doesn't always require treatment. The total knowledge score indicated a moderate level of knowledge among PHPs, with a distribution across good, moderate, and poor knowledge levels. In managing acute gout, PHPs showed a preference for multiple interventions, but not all chose the recommended joint aspiration [9]. Knowledge of effective acute management strategies (NSAIDs, colchicine, and steroids) was sound. This aligns with the 2022 study conducted in the Qassim region, indicating that PHPs are well-versed in managing acute episodes [7]. However, less than half of the PHPs correctly identified when to initiate urate-lowering therapy and the duration of anti-inflammatory prophylaxis, suggesting a knowledge gap in the long-term management of the condition. This is similar to the findings of the study conducted in Jeddah, where nearly half of the participants incorrectly prescribed urate-lowering medication [6].

A significant majority understood the importance of diet and lifestyle modifications. There were no gender-based differences in knowledge and practice scores. Family medicine practitioners scored higher in knowledge and practices compared to general practitioners. Years of experience did not significantly impact knowledge or practice scores. Attending CME or reading about AH or gout in the past year did not significantly influence knowledge or practice scores. In contrast to this, Liu et al. [9] indicate that attending CME programs on AH or gout has a positive impact. They emphasized the need for quality CME to improve the management of gout among general practitioners [9]. Furlan et al. (2021) reported that PHPs who read scientific papers and researched for quality CME on hyperuricemia in the past year had significantly higher knowledge scores [10]. In our study, participants who attended CME scored an average of 5.36 with a standard deviation of 1.24. In contrast, those who did not attend CME scored an average of 5.08 with a standard deviation of 1.50. The statistical analysis (Z=1.127; p=0.260) suggests that the difference in scores between the two groups is not significant. Similarly, Liu et al. [9] found that general practitioners who had a better understanding of the basic concepts related to gout tended to have higher knowledge and better management of gout. They also emphasized the role of CME in improving the understanding of gout diagnosis and treatment among healthcare professionals; awareness of guidelines positively correlated with higher knowledge scores [9].

The PHPs generally demonstrated a moderate level of knowledge and practice in managing AH and gout. This suggests a good foundation but also highlights areas for improvement, especially regarding the understanding of certain disease aspects and management strategies. The findings suggest that specialty training (family medicine vs. general practice) has a more significant impact on knowledge and practice levels than gender, age, or years of experience. This emphasizes the importance of specialized training and targeted educational interventions in primary care settings. The lack of a significant difference in knowledge and practice scores between PHPs who attended CME and those who didn't could indicate either the ineffectiveness of current CME programs or the potential for other factors influencing knowledge and practice levels. The significant correlation between guideline awareness and higher knowledge scores underlines the importance of disseminating and ensuring access to updated clinical guidelines among PHPs. This study suggests that PHPs possess a reasonable level of knowledge and practice skills in managing AH and gout, with room for improvement. Specialized training and guideline awareness appear crucial in enhancing PHPs' capabilities. Continuous medical education, in its current form, may need evaluation and improvement to make a more significant impact. This study underscores the need for ongoing education and support for PHPs, particularly in areas of emerging or complex medical knowledge.

Limitations

Although there were a significant number of participants in conducting good research, it is still limited to the Asir region. There could be a potential self-reporting bias. It’s a cross-sectional design that doesn't capture changes over time and focuses on a single region. The study also lacks an exploration of the reasons behind knowledge and practice gaps. Despite these limitations, it provides valuable insights into PHPs' knowledge and practices related to AH and gout. It underscores the need for ongoing education and support for healthcare providers to improve patient care in this context. Future research should address these limitations and focus on enhancing patient outcomes in AH and gout management.

## Conclusions

In conclusion, our study highlights the need for continuous medical education and training programs to bridge the identified gaps in knowledge and practices among PHPs. Future research should focus on understanding the barriers to optimal management of AH and gout and developing strategies to overcome them. This will ultimately contribute to improved health outcomes for individuals with AH and gout, in line with the study's aim.
